# The Clinical Impact of CLIR Tools toward Rapid Resolution of Post-Newborn Screening Confirmatory Testing for X-Linked Adrenoleukodystrophy in California

**DOI:** 10.3390/ijns6030062

**Published:** 2020-08-05

**Authors:** Hao Tang, Jamie Matteson, Piero Rinaldo, Silvia Tortorelli, Robert Currier, Stanley Sciortino

**Affiliations:** 1 Genetic Disease Screening Program, California Department of Public Health, Richmond, CA 94804, USA; jamie.matteson@cdph.ca.gov (J.M.); stanley.sciortino@cdph.ca.gov (S.S.); 2 Biochemical Genetics Laboratory, Department of Laboratory Medicine and Pathology, Mayo Clinic, Rochester, MN 55905, USA; rinaldo@mayo.edu (P.R.); tortorelli.silvia@mayo.edu (S.T.); 3 Department of Pediatrics, University of California, San Francisco, CA 94158, USA; robert.currier@ucsf.edu

**Keywords:** newborn screening, X-linked adrenoleukodystrophy, very long-chain fatty-acids, ATP binding cassette subfamily D member 1, variants of uncertain significance, confirmatory test, bioinformatics, post-analytical interpretation, screening test performance

## Abstract

Since the start of X-linked adrenoleukodystrophy (ALD) newborn screening in California, more than half of the diagnosed cases were found to have an ATP binding cassette subfamily D member 1 (*ABCD1*) gene variant of uncertain significance (VUS). To determine retrospectively the likelihood that these were true positive cases, we used a web-based post-analytical tool in Collaborative Laboratory Integrated Reports (CLIR). Confirmatory plasma very long-chain fatty-acids (VLCFA) profiles for ALD screen positive infant boys were run through the CLIR ALD tool. We compared the distribution by *ABCD1* variant classification (pathogenic, likely pathogenic, VUS, and no variant) with the CLIR tool score interpretation (non-informative, possibly ALD, likely ALD, and very likely ALD) and the current case diagnosis. The study showed that CLIR tool positive interpretations were consistent with 100% of the pathogenic and likely pathogenic variants on the *ABCD1* gene if a more conservative guideline was used. The tool interpretations were also consistent with screened cases that were determined to not have disease (our no-disorder group). The CLIR tool identified 19 diagnosed ALD cases with VUS to be potential false positives, representing a 40% reduction among all diagnosed ALD cases with VUS. The reduction could be extended to 65% if a more aggressive threshold was used. Identifying such preventable false positives could alleviate the follow-up burden for patients, their families, and California Special Care Centers.

## 1. Introduction

X-linked adrenoleukodystrophy (ALD) is the most common inherited peroxisomal disorder, which is caused by variants in the X chromosome ATP binding cassette subfamily D member 1 (*ABCD1*) gene [1,2]. Mostly manifested among males, the disorder can lead to increased concentrations of very long-chain fatty acids (VLCFA) in plasma, as well as in adrenal and nervous tissues, affecting the adrenal cortex and the central nervous system with a wide range of clinical phenotypes that include adrenal insufficiency, adrenomyeloneuropathy (AMN), and its most severe form, childhood cerebral ALD [3,4,5]. These specific phenotypes cannot be predicted by *ABCD1* variant, concentrations of VLCFA, or family history [6,7]. Cerebral ALD symptoms with progressive and rapid neurologic decline may present between 2.5 and 10 years of age and can lead to death, severe disability, or a vegetative state within two to four years of onset in the absence of early treatment [5]. There is increasing evidence that hematopoietic stem cell transplantation (HSCT) can be an effective therapy to slow or halt cerebral demyelination and prevent death if administered in the early stage of the disease onset, indicating the potential benefits of newborn screening [8,9,10].

On 16 February 2016, ALD was officially added to the Recommended Uniform Screening Panel (RUSP) after testing methods were shown to be viable for newborn screening with high sensitivity and throughput [11,12,13,14,15]. California became the third state to universally screen for ALD on 21 September 2016, with retrospective screening of the specimens accessioned on or after 16 February 2016 following the amended state mandate [16,17].

In California, a three-tiered approach is employed to perform ALD screening on dried blood spots (DBS). First, C26:0-lysophosphatidylcholine (C26:0-LPC) is analyzed using flow injection analysis tandem mass spectrometry (FIA-MS/MS). Specimens with C26:0-LPC above the first-tier cutoff are further analyzed using liquid chromatography tandem mass spectrometry (LC-MS/MS) to obtain a more precise measurement (second tier). For newborns found to have elevated C26:0-LPC at the second tier, their DBS is sent for *ABCD1* variant analysis to complete ALD newborn screening.

Once the results of variant analysis are available, the California Genetic Disease Screening Program (GDSP) refers all ALD-positive cases to one of fourteen metabolic Special Care Centers (SCCs) across the state for clinical follow-up, which includes a diagnostic process based on screening results and other confirmatory testing. Plasma very long-chain fatty acid (VLCFA) analysis is the main confirmatory measure for ALD diagnosis [18,19,20], and has been integrated in other state ALD screening programs [14,21]. For the majority of ALD positive cases in California, the SCC will order plasma VLCFA to confirm the elevated C26:0-LPC found on newborn screening; they may also order additional confirmatory testing to differentiate an ALD diagnosis from other peroxisomal disorders (such as Zellweger spectrum disorder) or from a false positive. Confirmatory testing is not reimbursed by the California newborn screening program but is generally covered by insurance or other third-party payers.

In the first few years of ALD newborn screening in California, more than 40% of screen positive infants were found to have an *ABCD1* gene variant of uncertain significance (VUS), which may lead to more uncertain diagnosis of ALD cases [22]. In this study, we used a web-based post-analytical tool in Collaborative Laboratory Integrated Reports (CLIR) to examine confirmatory VLCFA profiles and evaluate retrospectively the potential reduction in diagnosed ALD cases with a VUS genotype.

## 2. Materials and Methods

### 2.1. Study Population and Sample

In California, all ALD testing results (biochemical and DNA sequencing), along with demographic information and follow-up report data (short-term follow-up to confirm a diagnosis and long-term clinical follow-up for up to 21 years) associated with the referred newborn are entered and stored in a web-based screening information system (SIS), including a newborn screening registry that houses all clinically diagnosed ALD cases. The categories of ALD disease case resolutions (for males) include: (1) ALD—not otherwise specified, (2) ALD—childhood cerebral, (3) ALD—Addison disease only, (4) ALD—AMN non cerebral, and (5) ALD—AMN cerebral. Carrier status (for females) and other types of peroxisomal disorders (for both males and females) such as Zellweger spectrum disorder (ZSD) are also recorded in the registry, but these cases are not assigned for long-term clinical follow-up. At the time of this analysis, none of the cases diagnosed through screening have shown clinical evidence to be reclassified as one of the more specific ALD disease categories; thus, all diagnosed cases are categorized as ALD—not otherwise specified. *ABCD1* variants are classified as pathogenic, likely pathogenic, and VUS, based on multiple existing variant and disease databases with published references including PubMed search [7,23,24]. Variants not meeting the criteria for being pathogenic or benign are classified as VUS based on the guideline from American College of Medical Genetics and Genomics and the Association for Molecular Pathology, including those with in silico analyses implying a potentially deleterious effect to the protein function [25]. Newborns with a positive screen and an *ABCD1* VUS are also considered “ALD—not otherwise specified” if plasma VLCFA interpretation is positive, and they receive long-term follow-up in our current protocol.

For this study, we selected California confirmatory testing data collected from 16 February 2016 through 31 October 2019 for this study’s population base. Cases included in the analysis were ALD screen-positive (C26:0-LPC elevation in second tier LC-MS/MS testing) male individuals with final resolutions including ALD—not otherwise specified, other peroxisomal disorders (including ZSD), and no disorder; and complete plasma total lipid VLCFA profiles that included C22:0-, C24:0-, C26:0-LPC, phytanic acid, and pristanic acid. For cases that had repeat plasma VLCFA confirmatory testing, we examined the latest testing results (and its corresponding diagnosis). VLCFA results were compiled in a comma-separated values (.csv) file inclusive of Logical Observation Identifiers Names and Codes (LOINC^®^) and covariates (age at the time of collection in years, sex) and were uploaded to CLIR for analysis by the tool runner functionality.

### 2.2. CLIR Tool

CLIR is a multivariate pattern recognition software and a web-based interactive analytical tool, funded, developed, and maintained by Mayo Clinic [26]. Initially developed to support laboratory quality improvement of newborn screening by MS/MS [27], CLIR now has analytical applications for a wide range of disorders that are detectable by newborn screening [26], but also includes applications for almost all biochemical genetic testing in a clinical setting. One of these CLIR applications is a tool that supports the biochemical diagnosis of peroxisomal disorders (POX) using serum VLCFA profiles. As of 20 February 2020, the POX ALD tool was constructed based on the VLCFA profile of 32,223 controls and 221 confirmed ALD cases with an age range between 1 day and 74 years, 33 of them with age <1 year. ALD-specific informative markers and calculated ratios are integrated in a tool that provides a score below or above a threshold of clinical significance and a likelihood of disease expressed as a percentile rank in comparison to known cases. A separate tool, named dual scatter plot, provides on demand differential diagnosis between ALD and a group of 11 other conditions, inclusive of peroxisomal disorders (7 conditions) and acquired artifacts (i.e., non-fasting, ketogenic artifacts). In the present study, C22:0-, C24:0-, C26:0-LPC, phytanic acid, and pristanic acid were used in the tool.

Authorized users of the ALD tool upload their own VLCFA test results file, run the tool, and receive either non-informative scores (no disorder) or informative scores, further broken down as possibly ALD, likely ALD, and very likely ALD. In the default ALD tool interpretation guideline, scores of less than 5 are considered not informative; scores equal to or over 5 and less than 125 are considered possibly ALD; scores equal to or over 125 and less than 290 are considered likely ALD; and scores equal to or over 290 are considered very likely ALD. In our study, we used the default ALD tool guideline to interpret the results, except that we considered a score of 0 to be non-informative, and anything above 0 as an informative score to get a more conservative estimate.

### 2.3. Analysis

We examined the CLIR ALD tool run score distribution categorized by *ABCD1* variant classification with a box and whisker plot as the basis for a sensitivity study (whether the CLIR ALD tool missed any ALD cases with pathogenic variants). A cross-tabulation analysis was performed to compare CLIR tool run result interpretations (non-informative, possibly ALD, likely ALD, and very likely ALD) to current diagnosis of ALD screen positive individuals with VUS. To estimate likely reductions of ALD diagnoses among screen-positive infants as a whole, we compared the current ALD case count and birth prevalence to the hypothetical case count and prevalence if VUS with non-informative CLIR ALD tool run results were considered no-disorder. A similar hypothetical case count and prevalence was estimated by using an interpretation guideline that considered VUS with a score equal to or less than 5 as non-informative to explore the impact of a more aggressive threshold to reduce potential ALD false positives in newborns with *ABCD1* VUS.

All analyses were performed with SAS/STAT software version 9.4 of the SAS system for Windows (SAS Institute, Cary, NC, USA).

## 3. Results

In the study period, 146 male infants were determined to be ALD screen positive, including 88 infants who had an ALD diagnosis. Of the 146 ALD positives, 112 met the inclusion criteria for the study, including 70 infants who had an ALD diagnosis.

**Figure 1:**
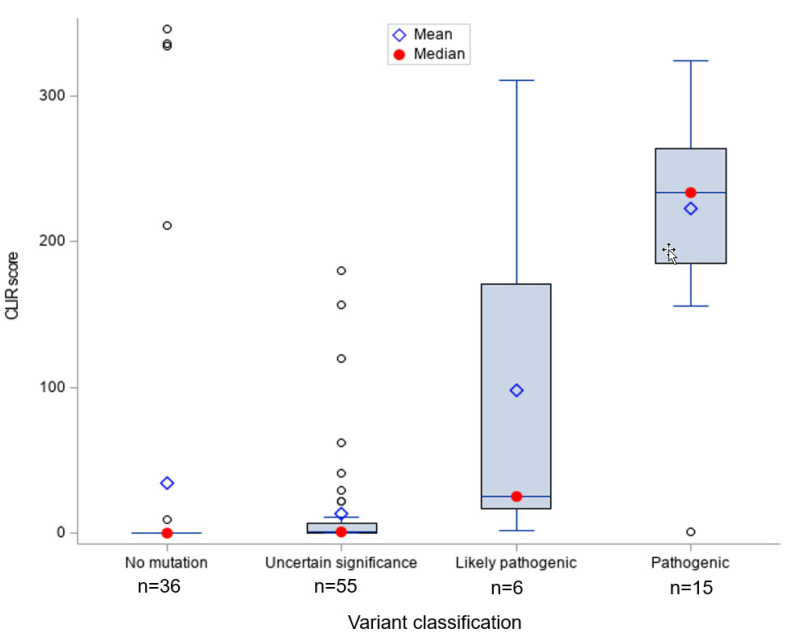
Collaborative Laboratory Integrated Reports (CLIR) peroxisomal disorders (POX) adrenoleukodystrophy (ALD) score distribution by *ABCD1* variant classification. Note: Each o represents an outlier data point.

Figure 1 shows that infants with *ABCD1* pathogenic (*n* = 15) and likely pathogenic (*n* = 6) variants had significantly higher CLIR tool run scores compared to ALD positive infants with other types of variants, indicating the CLIR tool informative interpretation was associated with all cases with a pathogenic or likely pathogenic variant. Only one case with a pathogenic variant scored below 100 (score = 1). We reviewed the newborn screening results and SCC short-term follow-up clinical case notes for this case and confirmed that the patient had a pathogenic variant previously reported in multiple individuals with ALD. 

The vast majority of the ALD positives without a variant (*n* = 36) had a tool run score of 0; the few exceptions with high scores (>200) had the diagnosis of ZSD. The one case with a low score and a possibly ALD interpretation was clinically diagnosed as ALD without an *ABCD1* variant or deletion, with specialist’s notes stating that: (1) The infant might have a non-coding *ABCD1* variant not detectable by available tests, (2) the case could be a false positive, and (3) the infant might have other peroxisomal disorder.

Most ALD positives with a VUS (*n* = 55) had very low tool run scores, with a few exceptions. A comparison of CLIR ALD tool interpretations and current diagnoses for screen positive individuals with VUS is shown in Table 1. Of the 112 ALD positive cases included in the study, half had an *ABCD1* VUS (*n* = 55, 49.1%). Nearly half (*n* = 25, 45.5%) of these ALD positive cases with VUS received a non-informative score for their initial plasma VLCFA test results, 50.9% (*n* = 28) of the VUS cases were interpreted as possibly ALD by the CLIR tool run results, and two were considered as likely ALD.

Currently in California, ALD screen positive infants with a VUS are diagnosed with ALD if plasma VLFCA confirmatory testing is interpreted as positive by confirmatory testing labs and clinicians. Of the 55 ALD positive infants with VUS, only four had normal plasma VLCFA results and were resolved as no disorder. Three other diagnoses were changed to no-disorder after repeat confirmatory test results were found to be normal. The remaining 48 were diagnosed as ALD—not otherwise specified, representing 68.5% of all diagnosed ALD cases with a complete confirmatory VLCFA profile (*n* = 70). 

With the CLIR tool run, 19 of the 48 diagnosed ALD cases had a non-informative score. In other words, 39.6% of diagnosed ALD infants with VUS would not be considered to have ALD based on the CLIR ALD tool interpretation. Among the 48 diagnosed ALD cases with VUS, only 2 VUS cases were identified as likely ALD, and the other 27 were possibly ALD (29 in total). None of the ALD screen positives with VUS were considered by the CLIR tool as very likely ALD. If we make the threshold of the non-informative category equal to or less than 5 for screen-positive infants with a VUS, then the number of diagnosed ALD individuals with a VUS and with an informative CLIR tool score could be reduced further from 29 to 17 (data not shown). In other words, 31 out of 48 individuals with a current ALD diagnosis and *ABCD1* VUS would be considered false positive, representing a 64.6% reduction in diagnosis for that group.

Table 2 shows the potential impact on all ALD positives when using CLIR ALD tools to review VLCFA results. The 19 currently diagnosed ALD cases that were considered non-ALD by CLIR tools were exclusively from screen positives with VUS. Of the 37 ALD positive infants resolved as no disorder, 36 received a score of zero from the CLIR ALD tool run. A total of 56 ALD screen positive infants had an informative CLIR ALD tool score, including 51 currently diagnosed ALD cases and 4 cases diagnosed as other peroxisomal disorders.

In addition to the 70 diagnosed cases with a complete plasma VLCFA profile, there were 18 diagnosed cases that did not have a complete VLCFA profile (data not shown). The total number of cases resolved as ALD (88) through newborn screening represents an estimated birth prevalence of 1 in 9995. Using CLIR as an evaluation tool for VLCFA results, the total number of ALD cases would drop from 88 to 69 (51 with a complete plasma VLCFA profile and 18 without) and the birth prevalence would decrease to 1 in 12,747 (data not shown).

If we used the more aggressive threshold for the non-informative category (≤5) for positive infants with VUS, then the total number of diagnosed ALD individuals would be 57 (39 with a complete plasma VLCFA profile and 18 without; data not shown), representing an estimated birth prevalence of 1 in 15,430 (data not shown).

## 4. Discussion

Since the start of ALD newborn screening in California, a larger proportion of screen positive infant boys have been found to have a VUS on the *ABCD1* gene, compared to the reports from other state programs [21,28]. They have been considered to have ALD if the plasma VLCFA confirmatory test is positive, which is the case for most of them. To study the potential reduction of ALD diagnoses among screen positive males with an *ABCD1* VUS, we used an ALD—specific analytical tool to re-examine the plasma VLCFA confirmatory test interpretations. We found that the CLIR POX ALD tool-run results were accurately linked to 100% of the pathogenic and likely pathogenic variants on the *ABCD1* gene. The tool run results also confirmed all the current no-disorder cases based on *ABCD1* variant analysis and previous plasma VLCFA interpretation. Furthermore, the CLIR tool was able to identify 19 individuals with VUS currently diagnosed as ALD to be potential false positives, which would result in a 37.5% reduction in that group. The reduction could be extended to over 60% if a more aggressive informative threshold was used. Without any reduction in diagnosed ALD cases with VUS, the prevalence of ALD in California screening population was about 1 in 10,000, higher than that reported in the earlier literature (1 in 21,000) and in New York (1 in 16,074) [2,28], although significantly lower than that reported by Minnesota [21]. With a more aggressive interpretation algorithm when using the CLIR POX tool, the reduction in ALD cases would lead to a prevalence that was similar to the prevalence reported by the New York newborn screening program.

A conservative second-tier C26:0-LPC cutoff (≥0.15) instituted in the beginning of California ALD newborn screening is a possible explanation for the large number of screen positives with VUS. After the cut-off increase in December 2017 to 0.22, the program has seen the number of diagnosed cases with VUS decrease considerably [29]. However, the screen positive rate after the cutoff change is still higher than that reported by New York [28]. A supplementary analysis showed that 10 diagnosed ALD infants with VUS would be false positive if the cutoff of 0.22 had been used throughout the study period.

In the California newborn screening program, the protocol for a child diagnosed with ALD is to provide long term follow-up annually until 21 years of age. These children require ongoing monitoring including multiple MRIs and endocrine evaluations each year. Thus, the diagnosis burden is very large on the patient, their families, the SCCs, and the NBS program that compensates SCCs for yearly annual patient summary data provided by the SCCs. The benefits of fewer false positives are obvious, especially for a later-onset disorder like ALD where disease monitoring needs to continue throughout the lifespan of the patient. Our analysis showed that the CLIR POX tool for ALD is a feasible option for clinicians to review plasma or serum VLCFA confirmatory test results and make diagnostic decision for screen-positive infants with *ABCD1* VUS.

In the current ALD diagnostic workup, interpretation of the confirmatory VLCFA profile is mostly based on observing the occurrence of individual biomarker elevation. Effort has been made to utilize multiple markers simultaneously as a stronger analytical solution with statistical technique, such as Moser’s function based on the concept of discriminant analysis [19], which is included in most VLCFA lab reports. The CLIR tool for ALD, similar to other tools in CLIR, is a regression-based multivariate analytical tool allowing adjustment for multiple patient characteristics. The tool has the capacity of examining up to seven biomarkers, including C26:1-LPC and C24:1-LPC in addition to the five markers used in this study. The tool-run results indicated that a fairly large number of positive VLCFA lab results for screen positive individuals with VUS could be false positives, but we may have to wait many years to know the definitive case status, if symptoms ever arise.

The effective application of a plasma VLCFA profile as a confirmatory test for ALD may also suggest a multi-marker approach to the initial newborn screening algorithm on DBS samples. To our knowledge, most newborn screening programs currently use C26:0-LPC as the sole marker for initial ALD screening, although other VLCFA markers may be available from the same throughput. CLIR has an ALD tool for DBS samples that uses C20:0-, C22:0-, C24:0-, and C26:0-LPC adjusted for multiple covariates (age at collection and birthweight) to provide an interpretation of the likelihood of being a potential ALD case. Running screening multi-marker results through that tool, especially for samples that have elevated C26:0-LPC, can reduce false positives to a near zero level. For example, a 10-plex assay CLIR tool that combines six lysosomal enzymes and four LPC-species applied to the screening of 82,850 newborns born in Kentucky between July 2018 and December 2019 led to performing the second-tier test in 289 cases (0.3%), of which 4 were informative. All four cases were males confirmed as ALD with a prevalence of 1:20,712 and a positive predictive value (PPV) and false positive rate (FPR) of 100% and 0%, respectively (S. Tortorelli et al. unpublished results).

Being a phenotypical late-onset disorder, ALD is intrinsically challenging for screening, diagnosis, management, and treatment of the patients. “Confirmed cases” with VUS are predictive in nature. Improving performance of newborn screening and diagnosis, and reducing unnecessary long-term follow-up, will clearly have a positive impact on the quality of life for affected children and their families. With more young patients receiving confirmation of disease status in the future, and more clinically confirmed case information integrating into the tool, screening and diagnostic analytical approaches such as CLIR ALD applications have the potential to produce more accurate interpretations. Even more important, by helping newborns receive a more accurate diagnosis through improved screening performance, the follow-up burden and emotional toll on families can be reduced by lowering the number of false positive cases.

## Figures and Tables

**Table 1:** Comparison of CLIR ALD tool run interpretation guideline results with current diagnosis for ALD screen positive individuals with variant of uncertain significance (VUS).

CLIR Tool Interpretation Guideline	Current Diagnosis		
	ALD—Not Otherwise Specified	No Disorder	Total
Non-informative	19	7	25
Possibly ALD	27	0	28
Likely ALD	2	0	2
Very likely ALD	0	0	0
Total	48	7	55

**Table 2:** Comparison of CLIR tool ALD interpretation with current diagnosis for all ALD screen positives.

CLIR Tool Interpretation	Current Diagnosis			
	ALD—Not Otherwise Specified	Other Peroxisomal Disorders (Including ZSD)	No Disorder	Total
ALD non-informative	19	1	37	56
ALD informative	51	4	0	56
Total	70	5	37	112
